# Risk Factors for High-Risk Adenoma on the First Lifetime Colonoscopy Using Decision Tree Method: A Cross-Sectional Study in 6,047 Asymptomatic Koreans

**DOI:** 10.3389/fmed.2021.719768

**Published:** 2021-09-23

**Authors:** Kwang Hyun Chung, Min Jung Park, Eun Hyo Jin, Ji Yeon Seo, Ji Hyun Song, Sun Young Yang, Young Sun Kim, Jeong Yoon Yim, Seon Hee Lim, Joo Sung Kim, Su Jin Chung, Joo Kyung Park

**Affiliations:** ^1^Division of Gastroenterology, Department of Internal Medicine, Uijeongbu Eulji Medical Center, Eulji University School of Medicine, Uijeongbu, South Korea; ^2^Department of Internal Medicine, Healthcare System Gangnam Center, Healthcare Research Institute, Seoul National University Hospital, Seoul, South Korea; ^3^Department of Internal Medicine, Sheikh Khalifa Specialty Hospital, Ras Al Khaimah, United Arab Emirates; ^4^Department of Internal Medicine and Liver Research Institute, Seoul National University College of Medicine, Seoul, South Korea; ^5^Division of Gastroenterology, Department of Internal Medicine, Samsung Medical Center, Sungkyunkwan University School of Medicine, Seoul, South Korea; ^6^Department of Health Sciences and Technology, Samsung Advanced Institute for Health Sciences and Technology, Sungkyunkwan University, Seoul, South Korea

**Keywords:** colonic polyps, colonic neoplasms, risk factors, mass screening (MeSH), decision tree method

## Abstract

**Background/Aims:** As risk of colorectal neoplasm is varied even in persons with “average-risk,” risk evaluation and tailored screening are needed. This study aimed to evaluate the risk factors of high-risk adenoma (HRA) in healthy individuals and determine the characteristics of advanced neoplasia (AN) among individual polyps.

**Methods:** Asymptomatic adults who underwent the first lifetime screening colonoscopy at the Seoul National University Hospital Healthcare System Gangnam Center (SNUH GC) were recruited from 2004 to 2007 as SNUH GC Cohort and were followed for 10 years. Demographic and clinical characteristics were compared between the subjects with and without AN (≥10 mm in size, villous component, and/or high-grade dysplasia and/or cancer) or HRA (AN and/or 3 or more adenomas). For individual polyps, correlations between clinical or endoscopic features and histologic grades were evaluated using a decision tree method.

**Results:** A total of 6,047 subjects were included and 5,621 polyps were found in 2,604 (43%) subjects. Advanced age, male sex, and current smoking status were statistically significant with regards to AN and HRA. A lower incidence of AN was observed in subjects taking aspirin. In the decision tree model, the location, shape, and size of the polyp, and sex of the subject were key predictors of the pathologic type. A weak but significant association was observed between the prediction of the final tree and the histological grouping (Kendall's tau-c = 0.142, *p* < 0001).

**Conclusions:** Advanced neoplasia and HRA can be predicted using several individual characteristics and decision tree models.

## Introduction

Colon cancer is the third most prevalent cancer and the second leading cause of cancer-related deaths worldwide (1). Like many other cancers, early detection is key to colorectal cancer treatment. Early stages of colon cancer usually require less extensive treatment and can result in better clinical outcomes (2, 3). Most colorectal cancers can be prevented by early detection and removal of precursor colorectal adenomas (3–6). Colonoscopy is one of the most sensitive and effective diagnostic modalities that can directly visualize colorectal lesions and remove premalignant adenomatous polyps or early cancers. However, colonoscopy requires a skilled examiner and is associated with significant costs, inconvenience, and procedure-related adverse events. These limitations of colonoscopy may decrease adherence to screening tests (7).

Current guidelines recommend that average-risk individuals start colorectal cancer screening at the age of 50 years unless they have the following risk factors: personal history of adenomatous polyps or colorectal cancer, family history of colorectal cancer, confirmed or suspected hereditary colorectal cancer syndrome (such as familial adenomatous polyposis or Lynch syndrome), personal history of abdominal or pelvic radiation for previous cancer, or personal history of inflammatory bowel disease (8, 9). However, there is surmounting evidence that the risk of colorectal neoplasm varies even in average-risk individuals. Precise evaluation of these risks may help to tailor colorectal cancer screening strategies and increase adherence to the screening program.

The aims of this large-scale cross-sectional study were to (1) evaluate the risk factors for advanced neoplasia (AN), and high-risk adenoma (HRA), and (2) determine the characteristics of polyps with advanced histology in healthy asymptomatic individuals from the first lifetime colonoscopy screening.

## Materials and Methods

### Study Population

Asymptomatic healthy adults who underwent screening colonoscopy at the Seoul National University Hospital Healthcare System Gangnam Center between October 2004 and June 2007 were recruited for participation in this study. To be included in the study, subjects were required to meet the following criteria: (1) first-lifetime screening colonoscopy, (2) asymptomatic volunteers aged over 20 years, and (3) complete clearing colonoscopy. A complete clearing colonoscopy was defined as colonoscope insertion into the cecum, above fair grade preparation with the Aronchick Bowel Preparation Scale (10), and removal of all detected polyps. In the case of a remnant polyp or poor grade preparation, the procedure was repeated within 6 months. All participants were requested to complete a structured questionnaire on gastrointestinal symptoms and medical histories. Further information was ascertained by endoscopists regarding the reasons for colonoscopy and prior diagnosis of colorectal polyps. Subjects who did not complete the questionnaire were excluded. Based on responses to the questionnaire, the following cases were excluded: colorectal disease-related symptoms or signs (e.g., recent bowel habit change, unexplained weight loss, anemia, and lower gastrointestinal tract bleeding not attributable to hemorrhoids), personal history of colorectal cancer or polyps, surgical resection of the colon or rectum, inflammatory bowel disease or intestinal tuberculosis, and coagulopathy which hinders endoscopic polyp resection.

The study protocol conformed to the ethical guidelines of the 1975 Declaration of Helsinki and its revisions and was approved by the Institutional Review Board of SNUH (No. 0709-025-218). Since the current study was performed with a retrospective design using a database and medical records, informed consent was waived by the board.

### Study Procedures and Definitions

Conventional white light colonoscopes (CF-H260 series; Olympus Co., Ltd., Aizu, Japan/ EC-450HL5, EC-450WM5, or EC-590ZW series; Fujinon Inc., Saitama, Japan) were used in all procedures. All colonoscopies were performed by 15 board-certified endoscopists who had experienced more than 5,000 colonoscopies (at least 500 polypectomies) and achieved an overall adenoma detection rate of over 30% in routine procedures.

Complete colonoscopy was defined as cecal endoscopic intubation with photo documentation of the appendiceal orifice. Colonoscopy reports provided information on the number, location, shape [according to the Paris classification (11)], and size (estimated with opened biopsy forceps or measured after resection) of the polyps. All detected polyps were completely removed. Diminutive polyps of <5 mm were removed using biopsy forceps, larger polyps were removed by endoscopic mucosal resection, and some very large polyps were removed by piecemeal endoscopic mucosal resection or endoscopic submucosal dissection. Specimens from all polyps were reviewed and a confirmatory diagnosis was made by two expert gastrointestinal pathologists, who classified colorectal neoplasms according to the WHO criteria (12). Findings were stratified by the most advanced lesion found (e.g., adenoma with the greatest diameter or the most serious histology). Serrated adenomas were excluded from the analysis. This is because there was no clear criterion for the size of serrated polyps. Further, there was no generally accepted definition for sessile serrated polyps during the period in which the actual endoscopic exam was performed, and an inconsistency in the diagnostic application for serrated adenoma between each pathologist was considered. Pathologic interpretation of intramucosal carcinoma or carcinoma in situ was categorized as high-grade dysplasia and non-neoplastic findings such as lipoma, lymphoid aggregates, or inflammatory polyps were considered normal mucosa and classified as “non-specific lesion” for histologic groups. AN was defined as an adenoma ≥10 mm, adenoma with tubulovillous or villous histology and, with high-grade dysplasia, or the presence of invasive cancer (13). HRA was defined as an AN or a case in which three or more adenomas were found in one person (13). To analyze the risk of AN in each polyp, each polyp was classified into four histologic groups: “non-specific lesion,” “hyperplastic polyp,” “non-advanced adenoma,” (including low-grade tubular adenoma), and “advanced neoplasia.”

### Assessment of Risk Variables

Structured self-administered questionnaires were reviewed for gastrointestinal symptoms and medical history including current smoking (smoked regularly during the previous 12 months), alcohol consumption (≥70 g/week), and regular consumption or use (i.e., medication for ≥3 months during the preceding 12 months) of aspirin or non-steroidal anti-inflammatory drugs (NSAIDs), 3-hydroxy-3-methylglutaryl CoA reductase inhibitor (statin), or hormonal replacement therapy. The questionnaires also asked about family history of any cancer including colorectal cancer (at least one first-degree relative with colorectal cancer diagnosed at any age), educational qualification, and monthly household income. Household income was divided into upper and lower classes based on $50,000 per year. Physical examinations for all subjects were performed on the day of colonoscopy by trained nurses using a written systematic protocol with standardized instruments. Body mass index (BMI) was calculated from measured weight and height, and categorized as normal (<23 kg/m^2^), overweight (23–24.9 kg/m^2^), or obese (≥25 kg/m^2^) according to the WHO Western Pacific Regional Office proposal (14). Waist circumference was measured at the WHO recommended site (midpoint between the lower border of the rib cage and iliac crest), and subjects whose waist circumference was ≥90 cm in men and ≥80 cm in women were classified as having central obesity according to the definition of the Asian population (15). All colonoscopy, pathology reports and medical records were collected from a database (Healthwatch version 2.0).

### Statistical Analysis

Continuous variables were expressed as mean ± standard deviation. Nominal and ordinal variables are stated as proportions and percentages. To compare the characteristics of individuals with and without AN or HRA, the chi-square test or Fisher's exact test was used for categorical variables, and Student's *t*-test was used for continuous variables after normal distribution was confirmed by performing the Anderson-Darling test. To identify the factors related to HRA or AN, subjects with or without HRA or AN were compared in subjects without a family history of colorectal cancer and adenoma and no positive findings in other tests as a univariate analysis. These variables included the following: age, sex, body mass index, waist circumference, smoking, alcohol, aspirin and/or NSAIDs, statin use, hormone replacement therapy, family history of colorectal cancer, education level, and household income. Variables found to be significant in univariate analysis were subsequently assessed using binary logistic regression with backward elimination method as a multivariate analysis. For each variable, the hazard ratio (HR) and 95% confidence interval (CI) were calculated. Differences were statistically significant when the two-tailed *p*-value was <0.05. R software (R for Windows V.4.0.2; The R Foundation for Statistical Computing, Vienna, Austria) was used for statistical analyses.

Decision tree analysis (16) was conducted to examine the factors associated with polyps, which are AN. As classification variables in the decision tree analysis, factors showing significant differences among the four histological groups mentioned above were considered. Then the final decision tree was estimated using the minimum value of the complexity parameter. The association between prediction from the final tree and histology grouping was checked with Kendall's tau-c and its 95% confidence interval. The decision tree analysis was supported by the Statistics and Data Center at Samsung Medical Center using the Recursive Partitioning and Regression Trees (rpart) package in R software (version 3.2.3).

## Results

### Clinical and Socioeconomic Characteristics of Subjects

**Figure 1:**
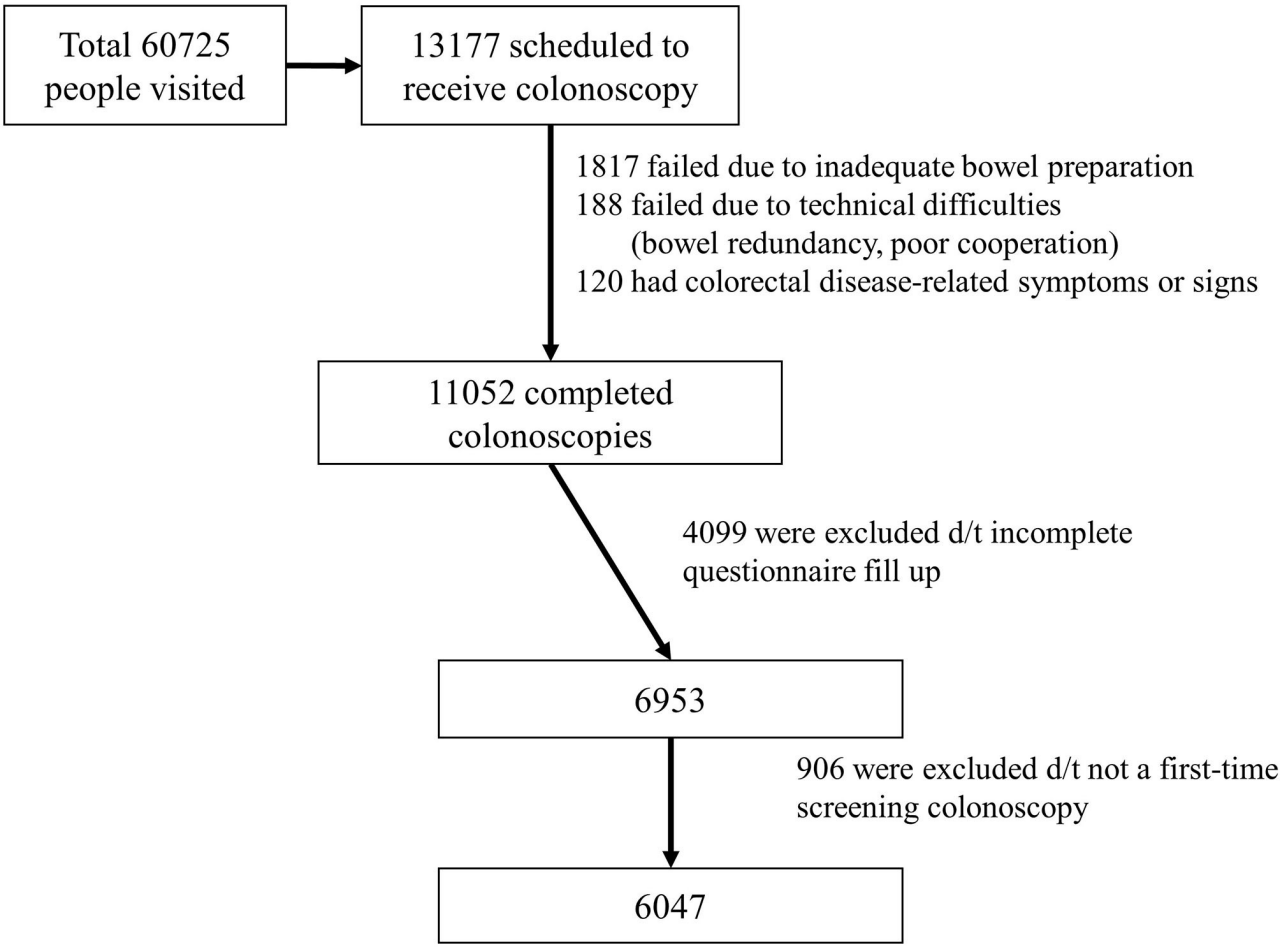
Flow chart of study enrollment.

During the study period, 60,725 people visited the institution of the researchers for routine health check-ups, and 13,177 patients were scheduled to undergo screening colonoscopy. Of these, 120 were excluded from the analysis because of colorectal disease-related symptoms or signs, 188 were excluded because the cecum could not be reached due to technical difficulties (bowel redundancy and/or poor cooperation), and 1,817 were excluded from the study due to inadequate bowel preparation. A number of 11,052 people completed screening colonoscopies and 4,099 people were excluded because of incomplete questionnaires. Of the 6,953 people remaining, 906 were excluded because they were not first-time screening colonoscopies. A total of 6,047 subjects who underwent the first lifetime colonoscopy were included and analyzed in this study (Figure 1).

The clinical and socioeconomic characteristics of the study population are described in Table 1. The study population included 5,294 subjects with no risk factors. Of these, 3,252 were over 50 years of age and 2,042 subjects were under 50 years of age. A total of 753 subjects showed some risk such as a family history of colorectal cancer or adenoma or had a positive result in other tests, of which 300 were under the age of 50 and 453 were over the age of 50.

**Table 1:** Baseline characteristics of the study population.

	**Age < 50 without** **risk factor**	**Age ≥ 50 without risk factor(Average risk population)**	**Age < 50 with** **risk factor**	**Age ≥ 50 with** **risk factor**	***P*-value**
***N***	2042	3252	300	453	
**Age group**					
<40	511 (25.0%)	0 (0.0%)	73 (24.3%)	0 (0.0%)	
40 to 49	1531 (75.0%)	0 (0.0%)	227 (75.7%)	0 (0.0%)	
50 to 59	0 (0.0%)	2022 (62.2%)	0 (0.0%)	303 (66.9%)	
60 to 69	0 (0.0%)	1057 (32.5%)	0 (0.0%)	129 (28.5%)	
70 or more	0 (0.0%)	173 (5.3%)	0 (0.0%)	21 (4.6%)	
**Sex**					<0.001
Female	688 (33.7%)	1237 (38.0%)	93 (31.0%)	129 (28.5%)	
Male	1354 (66.3%)	2015 (62.0%)	207 (69.0%)	324 (71.5%)	
**Known risk factor**					
No	2042 (100.0%)	3252 (100.0%)	0 (0.0%)	0 (0.0%)	
Polyp on sigmoidoscopy	0 (0.0%)	0 (0.0%)	81 (27.0%)	145 (32.0%)	
Polyp on CT colonography	0 (0.0%)	0 (0.0%)	44 (14.7%)	124 (27.4%)	
Positive on stool occult blood test	0 (0.0%)	0 (0.0%)	7 (2.3%)	1 (0.2%)	
Familial history of colorectal cancer	0 (0.0%)	0 (0.0%)	168 (56.0%)	183 (40.4%)	
**Body mass index (kg/m** ^**2**^ **)**					<0.001
Normal (<23)	871 (42.7%)	1214 (37.3%)	126 (42.0%)	161 (35.5%)	
Overweight (23–24.9)	497 (24.3%)	991 (30.5%)	76 (25.3%)	152 (33.6%)	
Obese (≥25)	674 (33.0%)	1047 (32.2%)	98 (32.7%)	140 (30.9%)	
**Waist circumference (cm)**					<0.001
<90 (male), <80 cm (female)	1267 (62.0%)	1591 (48.9%)	181 (60.3%)	220 (48.6%)	
≥90 (male), ≥80 cm (female)	775 (38.0%)	1661 (51.1%)	119 (39.7%)	233 (51.4%)	
**Current smoking**	550 (26.9%)	417 (12.8%)	76 (25.3%)	64 (14.1%)	<0.001
**Alcohol consumption**	416 (20.4%)	546 (16.8%)	59 (19.7%)	94 (20.8%)	0.005
**Family history of cancer other than colorectal cancer**	651 (31.9%)	848 (26.1%)	208 (69.3%)	251 (55.4%)	<0.001
**Hypertension**	188 (9.2%)	707 (21.7%)	22 (7.3%)	90 (19.9%)	<0.001
**Diabetes mellitus**	61 (3.0%)	281 (8.6%)	10 (3.3%)	41 (9.1%)	<0.001
**Medication use**					
Aspirin	71 (3.5%)	499 (15.3%)	8 (2.7%)	66 (14.6%)	<0.001
NSAIDs	22 (1.1%)	114 (3.5%)	2 (0.7%)	11 (2.4%)	<0.001
Statin	47 (2.3%)	239 (7.3%)	8 (2.7%)	35 (7.7%)	<0.001
Hormone replacement therapy	12 (0.6%)	160 (4.9%)	3 (1.0%)	7 (1.5%)	<0.001
**Education**					<0.001
Equal or Lower than high school	391 (19.1%)	1067 (32.8%)	52 (17.3%)	107 (23.6%)	
College or more	1651 (80.9%)	2185 (67.2%)	248 (82.7%)	346 (76.4%)	
**Household income**					<0.001
Middle class	421 (20.6%)	832 (25.6%)	54 (18.0%)	85 (18.8%)	
Upper class	1621 (79.4%)	2420 (74.4%)	246 (82.0%)	368 (81.2%)	

*NSAID, non-steroidal anti-inflammatory drugs*.

### Colonoscopic Features and Histopathologic Findings

**Figure 2:**
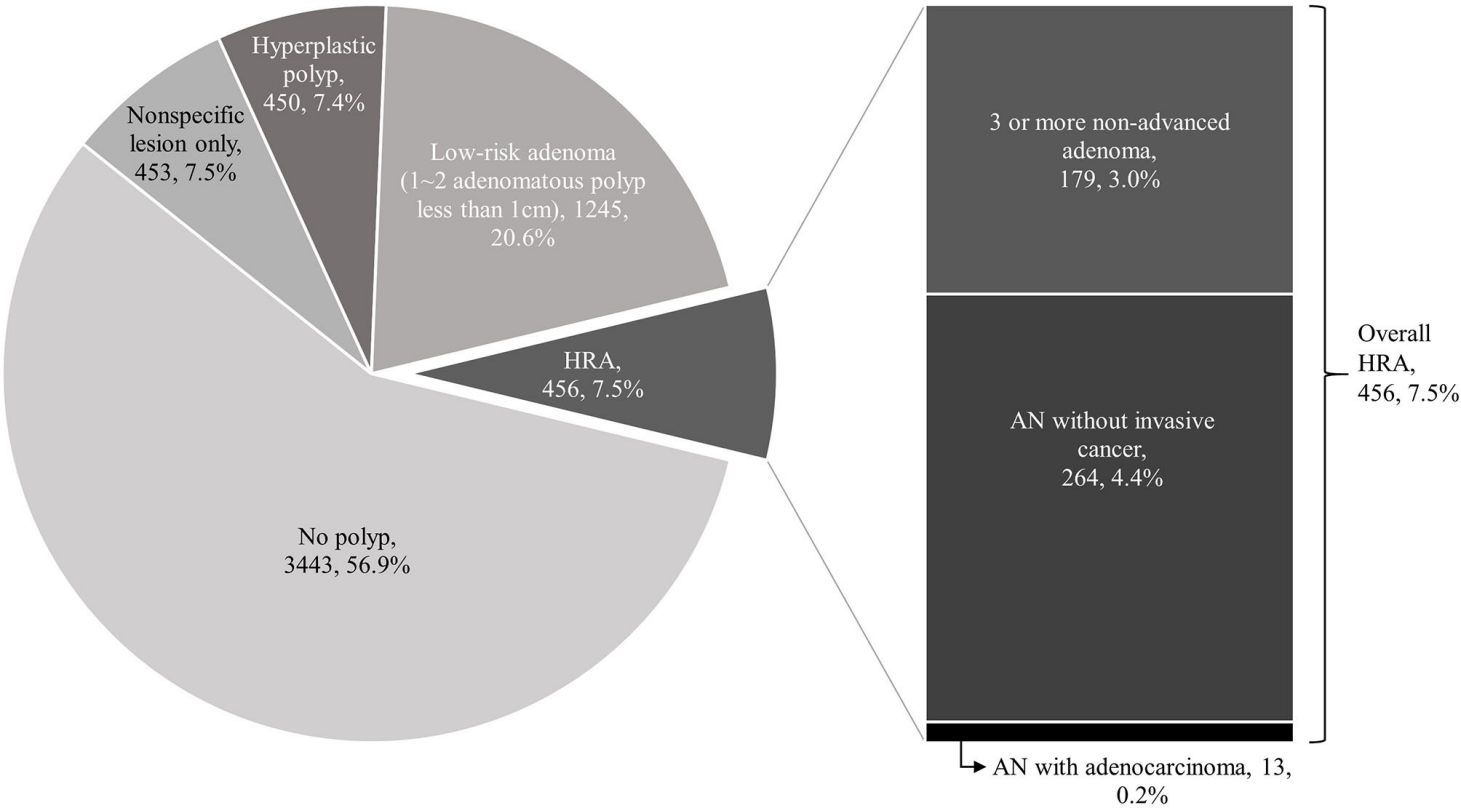
Incidence of advanced neoplasia (AN), high-risk adenoma (HRA) and adenocarcinoma. In a total of 6,047 subjects, 3,443 subjects did not have a colorectal polyp (57%). Among the rest of 2,604 subjects, 903 had only non-specific or hyperplastic polyps (15%), 1,245 (20.6%) had low-grade adenoma without high-risk feature, and 443 (7.3%) subjects were classified as who have HRA. Among the 443 subjects of HRA, 264 (4%) had AN and 13 (0.2%) had adenocarcinoma.

Of the 6,047 enrolled subjects, 1,245 (20.6%) had low-grade adenoma without high-risk features, and 456 (7.5%) subjects were classified as having HRA. Among the 456 subjects with HRA, 277 (4.6%) had AN and 13 (0.2%) had adenocarcinoma (Figure 2). The endoscopic and pathologic characteristics of polyps are shown in Table 2. Overall, 1,701 (28.1%) subjects had at least one adenoma, 1,435 (23.7%) subjects had one or two adenomas, and 266 (4.4%) subjects had three or more. Histologic features of the most advanced lesions were as follows: 1,555 (59.7%) subjects had low-grade adenomas, and 113 (4.3%) subjects had tubulovillous adenoma. Twenty (0.8%) subjects had high-grade dysplasia and 13 (0.5%) subjects had adenocarcinoma. In patients with colorectal neoplasm, the most advanced lesion was located in the ascending colon in 446 (26.2%) subjects, transverse colon in 494 (29.0%) subjects, descending colon in 182 (10.7%) subjects, sigmoid colon in 418 (24.6%) subjects, and rectum in 161 (9.5%) subjects. More than half of the most advanced lesions were ≤5 mm and 12.9% were ≥10 mm. The shape of the most advanced lesions was 0-Is (sessile) in 1,266 (74.5%) subjects, 0-Isp (subpedunculated) in 272 (16.0%) subjects, and 0-Ip (pedunculated) in 132 (7.8%) subjects.

**Table 2:** Colonoscopy findings and histopathologic results of polyps.

**Total subject number**	6047
**Number of polyps per subjects**	
0	3443 (56.9%)
1–2	1963 (32.5%)
≥ 3	641 (10.6%)
**Number of adenomas per subjects**	
0	4346 (71.9%)
1–2	1435 (23.7%)
≥3	266 (4.4%)
**Histologic features of most advanced lesion of each subject**	
Non-specific lesion	453 (17.4%)
Hyperplastic polyp	450 (17.3%)
Tubular adenoma	1555 (59.7%)
Tubulovillous/villous adenoma	113 (4.3%)
High-grade dysplasia	20 (0.8%)
Adenocarcinoma	13 (0.5%)
**Characteristics of the most advanced lesion in subjects with adenomatous polyp**	
*Location*	
Ascending colon	446 (26.2%)
Transverse colon	494 (29.0%)
Descending colon	182 (10.7%)
Sigmoid colon	418 (24.6%)
Rectum	161 (9.5%)
*Size*	
1–5 mm	974 (57.3%)
6–9 mm	507 (29.8%)
≥10 mm	220 (12.9%)
*Shape*	
0-Is (sessile)	1266 (74.5%)
0-Isp (subpedunculated)	272 (16.0%)
0-Ip (pedunculated)	132 (7.8%)
0-IIa,b,c (non-polypoid)	29 (1.7%)
Invasive cancer (mass or ulcerative cancer)	2 (0.1%)
**Total polyp number**	5618
**Histologic features of each polyp**	
Non-specific lesion	1492 (26.6%)
Hyperplastic polyp	1085 (19.3%)
Tubular adenoma	2882 (51.3%)
Tubulovillous adenoma	125 (2.2%)
High-grade dysplasia	21 (0.4%)
Adenocarcinoma	13 (0.2%)
**Endoscopic features of each polyp**	
*Location*	
Ascending colon	1351 (24.0%)
Transverse colon	1457 (25.9%)
Descending colon	545 (9.7%)
Sigmoid colon	1323 (23.5%)
Rectum	942 (16.8%)
*Size*	
1–5 mm	4243 (75.5%)
6–9 mm	1068 (19.0%)
≥10 mm	305 (5.4%)
*Shape*	
0-Is (sessile)	4781 (85.1%)
0-Isp (subpedunculated)	526 (9.4%)
0-Ip (pedunculated)	210 (3.7%)
0-IIa,b,c (non-polypoid)	99 (1.8%)
Invasive cancer (mass or ulcerative cancer)	2 (<0.1%)

### Factors Associated in Patients With AN or HRA

Univariate analysis of risk factors for AN and HRA was performed on subjects with no known risk factors (familial history of colorectal cancer or adenoma, positive on other screening modalities) and are described in Table 3. The mean age of subjects with AN or HRA were higher than the mean age of subjects without AN or HRA (52.0 ± 9.6 vs. 57.7 ± 8.8, *p* < 0.001 for AN, and 51.8 ± 9.5 vs. 58.2 ± 8.7, *p* < 0.001 for HRA). Male sex was a significant risk factor for both AN and HRA. Factors significantly increased the risk of HRA included current smoking, heavy alcohol consumption, family history of cancers other than colorectal cancer, hypertension. The use of NSAIDs or aspirin decreased the risk of HRA.

**Table 3:** Factors associated with AN or HRA according to univariate analysis.

	**Without AN**	**With AN**	***P*-value**	**Without HRA**	**With HRA**	***P*-value**
**No**.	5066	228		4921	373	
**Age (mean** **±** **SD) (years)**	52.0 ± 9.6	57.7 ± 8.8	<0.001	51.8 ± 9.5	58.2 ± 8.7	<0.001
**Sex (male)**	3200 (63.2%)	169 (74.1%)	0.001	3071 (62.4%)	298 (79.9%)	<0.001
**Body mass index (kg/m** ^**2**^ **)**			0.818			0.350
Normal (<23)	1992 (39.3%)	93 (40.8%)		1949 (39.6%)	136 (36.5%)	
Overweight (23–24.9)	1428 (28.2%)	60 (26.3%)		1384 (28.1%)	104 (27.9%)	
Obese (≥25)	1646 (32.5%)	75 (32.9%)		1588 (32.3%)	133 (35.7%)	
**Waist circumference (cm)**			0.643			0.989
<90 (male), <80 cm (female)	2731 (53.9%)	127 (55.7%)		2656 (54.0%)	202 (54.2%)	
≥90 (male), ≥80 cm (female)	2335 (46.1%)	101 (44.3%)		2265 (46.0%)	171 (45.8%)	
**Smoking**	914 (18.0%)	53 (23.2%)	0.057	868 (17.6%)	99 (26.5%)	<0.001
**Alcohol**	912 (18.0%)	50 (21.9%)	0.157	873 (17.7%)	89 (23.9%)	0.004
**Family history of cancer**	1442 (28.5%)	57 (25.0%)	0.289	1414 (28.7%)	85 (22.8%)	0.016
**Hypertension**	847 (16.7%)	48 (21.1%)	0.106	809 (16.4%)	86 (23.1%)	0.001
**Diabetes**	326 (6.4%)	16 (7.0%)	0.832	309 (6.3%)	33 (8.8%)	0.066
**Medication use**
Aspirin	553 (10.9%)	17 (7.5%)	0.124	518 (10.5%)	52 (13.9%)	0.049
NSAIDs	128 (2.5%)	8 (3.5%)	0.482	120 (2.4%)	16 (4.3%)	0.045
Statin	275 (5.4%)	11 (4.8%)	0.807	264 (5.4%)	22 (5.9%)	0.749
HRT	167 (3.3%)	5 (2.2%)	0.466	166 (3.4%)	6 (1.6%)	0.089
**Education (college or more)**	3675 (72.5%)	161 (70.6%)	0.574	3563 (72.4%)	273 (73.2%)	0.789
**Household income (upper class)**	3878 (76.5%)	163 (71.5%)	0.093	3771 (76.6%)	270 (72.4%)	0.072

*AN, advanced neoplasia; BMI, body mass index; CRC, colorectal cancer; F, female; HRA, high-risk adenoma; HRT, hormone replacement therapy; M, male; NSAID, non-steroidal anti-inflammatory drugs; SD, Standard deviation*.

In multivariate analysis, factors significantly increased the risk of AN were advanced age, male sex, and current smoking. Aspirin use is associated with decreased AN. Factors related to HRA included advanced age, male sex, and current smoking. However, aspirin or NSAIDs, HRT, and statin use were not related to HRA. Other factors that are expected to be related to advanced colorectal neoplasms such as obesity, individual component and/or presence or absence of metabolic syndrome, or alcohol consumption were not significantly related to AN or HRA. Socioeconomic status including education or household income did not relate to higher rates of AN and HRA (Table 4).

**Table 4:** Factors associated with AN or HRA according to multivariate analysis.

**Total 5,294 subjects**	**AN**		**HRA**	
**Variables**	**Hazard ratio (95% CI)**	***P*-value**	**Hazard ratio (95% CI)**	***P*-value**
Age	1.08 (1.06–1.09)	<0.001	1.08 (1.07–1.10)	<0.001
Sex (male)	1.52 (1.11–2.10)	0.010	2.05 (1.57–2.71)	<0.001
Smoking	1.63 (1.15–2.29)	0.005	2.01 (1.53–2.61)	<0.001
Family history of cancer other than CRC			0.81 (0.62–1.04)	0.109
Aspirin	0.40 (0.23–0.64)	<0.001	0.79 (0.57–1.08)	0.158

*AN, advanced neoplasia; CRC, colorectal cancer; HRA, high-risk adenoma*.

### Prediction of the Histological Findings of Individual Polyps

Differences in the histology of polyps according to demographic and endoscopic features are described in Table 5. The age of subjects tended to be higher in accordance with pathologic grade (55.2 to 57.9, *p* < 0.001). Polyps in the proximal colon tended to be more adenomatous polyps (non-advanced adenoma + AN) (61.8 vs. 35.8%), but the proportion of AN was higher in the rectosigmoid colon (2.0 vs. 4.1%). The polyp size tended to increase as the pathologic grade advanced. Compared with hyperplastic polyps, adenomatous polyps tended to be more pedunculated (Isp to Ip), and polyps with AN were even more common.

**Table 5:** Clinical and endoscopic factors associated with advanced neoplasia (AN).

	**Non-specific lesion**	**Hyperplastic polyp**	**Non-advanced adenoma**	**Advanced neoplasia**	***P*-value**
**Age**	55.2 ± 8.9	54.1 ± 9.0	56.8 ± 8.9	57.9 ± 9.2	<0.001
**Sex**					<0.001
Male	1164 (25.8%)	907 (20.1%)	2326 (51.6%)	109 (2.4%)	
Female	328 (29.5%)	178 (16.0%)	556 (50.0%)	50 (4.5%)	
**Indication**					0.003
Routine screening	1350 (26.8%)	994 (19.7%)	2546 (50.5%)	149 (3.0%)	
Positive on other modality	142 (24.5%)	91 (15.7%)	336 (58.0%)	10 (1.7%)	
**Location**					<0.001
Proximal	878 (26.2%)	337 (10.1%)	2072 (61.8%)	66 (2.0%)	
Rectosigmoid	614 (27.1%)	748 (33.0%)	810 (35.8%)	93 (4.1%)	
**Size**					<0.001
1–5 mm	1326 (31.3%)	892 (21.0%)	2004 (47.2%)	21 (0.5%)	
6–9 mm	134 (12.5%)	166 (15.5%)	726 (68.0%)	42 (3.9%)	
≥10 mm	32 (10.5%)	27 (8.9%)	152 (49.8%)	94 (30.8%)	
**Shape**					<0.001
Flat	33 (33.3%)	24 (24.2%)	40 (40.4%)	2 (2.0%)	
Is	1348 (28.2%)	995 (20.8%)	2393 (50.1%)	45 (0.9%)	
Isp	75 (14.3%)	59 (11.2%)	329 (62.5%)	63 (12.0%)	
Ip	36 (17.1%)	7 (3.3%)	120 (57.1%)	47 (22.4%)	
Mass	0 (0%)	0 (0%)	0 (0%)	2 (100.0%)	
**Body mass index (kg/m** ^**2**^ **)**					0.002
Normal (<23)	437 (24.9%)	329 (18.7%)	919 (52.4%)	70 (4.0%)	
Overweight (23–24.9)	448 (26.3%)	315 (18.5%)	901 (52.9%)	40 (2.3%)	
Obese (≥25)	607 (28.1%)	441 (20.4%)	1062 (49.2%)	49 (2.3%)	
**Waist circumference (cm)**					0.007
<90 (male), <80 cm (female)	757 (25.2%)	625 (20.8%)	1544 (51.3%)	83 (2.8%)	
≥90 (male), ≥80 cm (female)	735 (28.2%)	460 (17.6%)	1338 (51.3%)	76 (2.9%)	
**Current smoking**					<0.001
No	1108 (26.5%)	719 (17.2%)	2218 (53.1%)	130 (3.1%)	
Yes	384 (26.6%)	366 (25.4%)	664 (46.0%)	29 (2.0%)	
**Alcohol consumption**					0.002
No	1132 (26.9%)	767 (18.2%)	2185 (51.9%)	128 (3.0%)	
Yes	360 (25.6%)	318 (22.6%)	697 (49.6%)	31 (2.2%)	
**Family history of colorectal cancer**					0.048
No	1394 (26.5%)	1005 (19.1%)	2713 (51.6%)	142 (2.7%)	
Yes	98 (26.9%)	80 (22.0%)	169 (46.4%)	17 (4.7%)	
**Family history of cancer other than colorectal cancer**					0.122
No	1011 (25.9%)	746 (19.1%)	2042 (52.3%)	105 (2.7%)	
Yes	481 (28.1%)	339 (19.8%)	840 (49.0%)	54 (3.2%)	
**Hypertension**					0.2
No	1196 (26.4%)	898 (19.8%)	2315 (51.1%)	123 (2.7%)	
Yes	296 (27.3%)	187 (17.2%)	567 (52.2%)	36 (3.3%)	
**Diabetes mellitus**					0.094
No	1371 (26.9%)	968 (19.0%)	2619 (51.3%)	148 (2.9%)	
Yes	121 (23.6%)	117 (22.9%)	263 (51.4%)	11 (2.1%)	
**Medication use**					
Aspirin					0.012
No	1273 (26.4%)	941 (19.5%)	2461 (51.0%)	150 (3.1%)	
Yes	219 (27.6%)	144 (18.2%)	421 (53.1%)	9 (1.1%)	
NSAIDs					0.277
No	1458 (26.6%)	1064 (19.4%)	2801 (51.2%)	153 (2.8%)	
Yes	34 (23.9%)	21 (14.8%)	81 (57.0%)	6 (4.2%)	
Statin					0.598
No	1411 (26.7%)	1013 (19.2%)	2709 (51.3%)	148 (2.8%)	
Yes	81 (24.0%)	72 (21.4%)	173 (51.3%)	11 (3.3%)	
Hormone replacement therapy					0.229
No	1458 (26.4%)	1071 (19.4%)	2834 (51.4%)	155 (2.8%)	
Yes	34 (34.0%)	14 (14.0%)	48 (48.0%)	4 (4.0%)	
**Highest education**					0.133
Equal or Lower than high school	391 (27.5%)	250 (17.6%)	731 (51.5%)	48 (3.4%)	
College or more	1101 (26.2%)	835 (19.9%)	2151 (51.2%)	111 (2.6%)	
**Household income**					0.029
Middle class	351 (27.6%)	231 (18.1%)	642 (50.4%)	50 (3.9%)	
Upper class	1141 (26.3%)	854 (19.7%)	2240 (51.6%)	109 (2.5%)	

*NSAID, non-steroidal anti-inflammatory drugs*.

**Figure 3:**
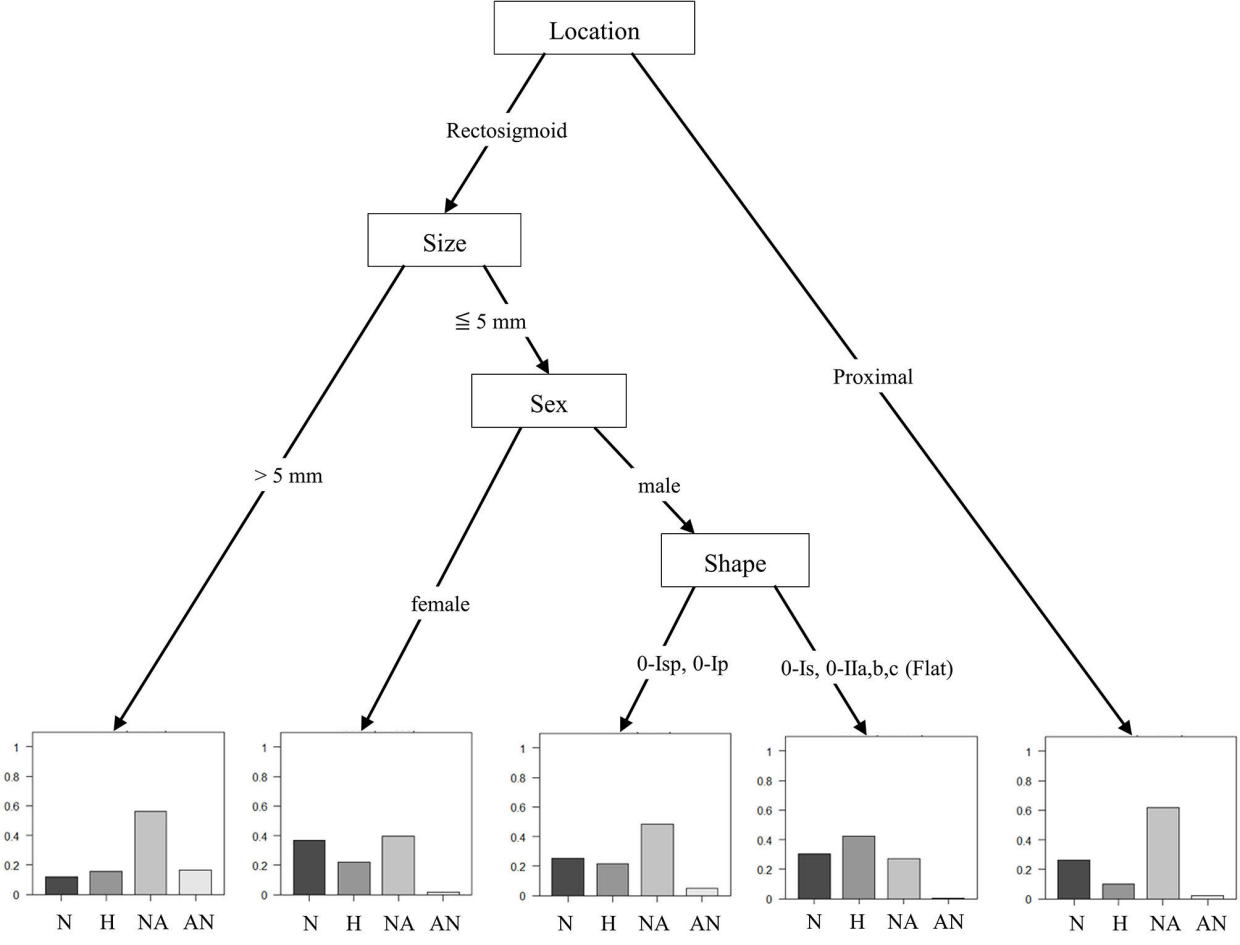
Decision tree model. According to the multivariate analysis by the decision tree analysis, location, size, and shape of polyp and the sex of subject were selected in the final decision tree with five lead nodes and considered to be significantly related with histology of polyp (Kendall's tau-c = 0.142, *p* < 0.0001). N, Non-specific lesion; H, Hyperplastic polyp; NA, Non-advanced adenoma; AN, advanced neoplasia.

According to the multivariate analysis performed via the decision tree analysis, location, size, sex, and polyp shape were selected in the final decision tree with five leaf nodes (Figure 3) and considered to be significantly related to polyp histology. Knowing the location of the polyp was the first step in predicting polyp histology and polyps >5 mm in the rectosigmoid area constituted the highest proportion of adenomatous polyps (72.9%) and AN (16.5%). Polyps <5 mm in the rectosigmoid area and polyps in female subjects revealed a low probability of adenomatous polyps (41.2%) and AN (1.5%). In male subjects, polyps shaped 0-Isp (subpedunculated) or 0-Ip (pedunculated) showed the second highest probability of being AN (5%). However, 0-Is (sessile) or flat polyps had the lowest probability of being AN (0.3%). In polyps located in the proximal colon, the probability of being a non-advanced adenoma was relatively high (63.8%), but the probability of being AN was low (2%). A significant but weak association was observed between prediction from the final tree and histology grouping (Kendall's tau-c = 0.142, *p* < 0.0001).

## Discussion

Population-based screening is a key strategy for improving colorectal cancer prognosis and can detect precursor adenomas or colorectal cancer at an early stage (8, 9, 17, 18). Colonoscopy screening for colorectal cancer reduces incidence and colorectal cancer related mortality (3, 6). Recent guidelines recommend that colorectal cancer screening in “average-risk subject” starts at age 50 and continues until 75 years of age since colorectal cancer was diagnosed most frequently in patients 65 to 74 years of age (18). However, there is evidence that the risk of colorectal neoplastic polyps and cancer varies among different risk groups (19–24). Therefore, colorectal cancer screening strategies need to be tailored and elaborated, to account for the diverse degree of risk in the individual person.

In this study, various demographic and clinical factors which could easily be gathered in daily medical practice were evaluated as risk factors for advanced colorectal neoplasms in asymptomatic subjects who underwent the first lifetime screening colonoscopy. Advanced age, male sex, and smoking were significant risk factors for both AN and HRA which is consistent with previous studies (19–22). Interestingly, in the present study, aspirin use decreased the risk of AN, but not HRA. Aspirin may have some protective effect on the progression of low-grade adenoma to AN but not in the development of low-grade adenoma. The prophylactic effect of aspirin is underrated in low-grade adenomas. Low-grade adenomas were classified as HRA with a number of 3 or more. Even though subjects with advanced colorectal neoplasms tended to have hypertension and diabetes, these factors were not statistically significant. Education level and household income were not statistically significant between the two groups, even though household income was slightly lower in subjects with advanced colorectal neoplasm.

Significant factors, which could predict the histology of individual polyps, were location, size, and shape of the polyp and the sex of the subject. In the model of the study, polyp location was the first and most important step in predicting histology. Proximal polyps had a high rate of adenomatous polyps. However, rectosigmoid polyps were also significant, especially those >5 mm in size and had more AN. Even in polyps located in the rectosigmoid area and had a size of ≤5 mm, attention is needed in Isp or Ip-shaped polyps of male subjects due to the high proportion of AN.

The distribution of adenomatous polyps within the colon is highly influenced by the characteristics of the study population, such as age or sex (25, 26). A previous large retrospective cohort study (27) and a multicenter retrospective cohort study in South Korea (28) showed that polyps in the proximal colon are more likely to be adenomatous polyp than distal polyps, which is consistent with the results. With regard to advanced adenoma, some studies have shown a similar ratio of advanced adenoma (29, 30) between the proximal and rectosigmoid colon. Another study reported that polyps >5 mm in the rectosigmoid colon are more likely to have advanced adenoma than in the proximal colon (31) which corresponds to the findings of the study. Although adenomatous polyps are more common in the proximal colon, polyps in the rectosigmoid colon should also be investigated because adenomatous polyps in the rectosigmoid colon can be more advanced.

It is already known that a larger polyp size is related to AN (29, 31–36). In the model of the study, size is an important step in determining histologic grade in rectosigmoid polyps. Most previous studies have shown a marked increase in histologic grade at 1 cm cut off (33, 34, 36) which is one of the criteria for AN (37, 38). However, the model shows a cutoff of 5 mm was significant for differentiating each node. In addition, polyps <5 mm account for 11.4% of polyps with high-grade dysplasia or carcinoma.

The shape of the polyp was also an important factor for discriminating histological features in male subjects with a size <5 mm. The 0-Isp or 0-Ip type polyps were more likely to be adenomatous or AN than 0-Is polyps or flat lesions. It seems that pedunculated polyps are more likely to be adenomas or ANs than sessile polyps which is consistent with a previous study (33).

In Korean epidemiologic studies, the incidence of rectal adenoma is similar to that of the proximal colon and distal colon adenoma, but advanced polyps are found more frequently in the rectosigmoid colon, and rectal cancer is more common than proximal colon cancer (39, 40); thus, rectal polyps should not be overlooked in clinical practice. This fact is consistent with the findings and suggests that even diminutive (<5 mm) polyps found in the rectosigmoid area should not be taken lightly.

In the study, the adenoma detection rate was relatively low at 28.1% (1,047/6,047) in all subjects, and 28.7% (1,517/5,294) in subjects without previously known risk factors. This may be because many of the included subjects were under 50 years of age. In subjects without risk factors, the adenoma detection rate by age was 17.6% (359/2,042) for those under 50, 29.9% for those in their 50s (604/2,022), 35.6% (376/1,057) for those in their 60s, and 51.4% (89/173) for those over 70 years old. However, adenomas are also found in patients younger than 50 years of age, albeit at a low rate, with AN in 2% of study subjects and HRP in approximately 3% of study subjects in this category. Therefore, screening colonoscopy may be necessary in some cases. According to previous reports, the incidence of adenomatous polyps increases with age (41, 42) and the study found that the proportion of adenomas and AN was higher in the older age group. However, age is not included as a significant factor in the decision tree model, likely due to its low effect. Alcohol consumption, metabolic syndrome (DM, HTN, high BMI, or abdominal obesity), medication use, education, and income were also considered but these factors were not related to an increased incidence of AN or HRP in the first lifetime screening colonoscopy.

The limitations of the study include the retrospective design, and the many demographic data were collected from patients' written reports which can be a source of recall bias. However, demographic features or socioeconomic status was investigated before the colonoscopy examination with a validated questionnaire that could minimize recall bias and drawbacks of retrospective design. On the other hand, there may be a risk of selection bias because a large number of subjects were excluded from the study at the beginning of the analysis. However, since most of the subjects were excluded because they did not fill out the questionnaire, and not because of differences in endoscopy results or specific factors, the risk of selection bias was considered to be negligible. Although many cases were excluded, many cases remained which was considered sufficient to answer the research questions. Additionally, serrated adenoma was inevitably excluded, and since this study was conducted at a single institution, there may be some limitations in generalizing the results of this study. Other various optical evaluation methods, such as pit pattern or narrow band image could not be used to distinguish the characteristics of polyps. In the study, a large number of subjects solely within the Asian population were included. All subjects were asymptomatic, and the colonoscopy was a first life time screening which is representative of the main target for screening colonoscopy, the general healthy population. Although this was a retrospective study, the prospective cohort was used, and many factors (e.g., medication use, household income, education) that might affect the incidence of colorectal polyps could be evaluated. The decision tree model in this study can be a useful tool for estimating the probability that each polyp is an AN and might be informative for whom screening colonoscopy is performed.

In conclusion, advanced age, male sex, family history of cancer other than colorectal cancer, and smoking may be risk factors for both AN and HRA in the first life time colonoscopy, and aspirin use may be protective factors for AN but not for HRA. The probability of AN in individual polyps could be predicted with the decision tree model of the study. It was found that the important factors in predicting AN were location, size, and shape of the polyp, and the sex of the subject. Identifying such risk factors in an average individual may help in making tailored decisions in clinical practice.

## Data Availability Statement

The raw data supporting the conclusions of this article will be made available by the authors, without undue reservation.

## Ethics Statement

The studies involving human participants were reviewed and approved by Seoul National University College of Medicine/Seoul National University Hospital Institutional Review Board (No. 0709-025-218). Written informed consent for participation was not required for this study in accordance with the national legislation and the institutional requirements.

## Author Contributions

KC, MP, JP, and SC contributed to the concept and design of the study. EJ, JYS, and JK critically reviewed the research protocol. EJ, JYS, JHS, SY, YK, JY, and SL collected, analyzed, and interpreted the data. KC performed the literature search and critically revised the manuscript. All authors contributed to drafting the manuscript and approved the final version.

## Conflict of Interest

The authors declare that the research was conducted in the absence of any commercial or financial relationships that could be construed as a potential conflict of interest.

## Publisher's Note

All claims expressed in this article are solely those of the authors and do not necessarily represent those of their affiliated organizations, or those of the publisher, the editors and the reviewers. Any product that may be evaluated in this article, or claim that may be made by its manufacturer, is not guaranteed or endorsed by the publisher.
